# Variation Among Spring Wheat (*Triticum aestivum* L.) Genotypes in Response to the Drought Stress. II—Root System Structure

**DOI:** 10.3390/plants8120584

**Published:** 2019-12-08

**Authors:** Maciej T. Grzesiak, Natalia Hordyńska, Anna Maksymowicz, Stanisław Grzesiak, Magdalena Szechyńska-Hebda

**Affiliations:** 1 F. Górski Institute of Plant Physiology, Polish Academy of Sciences, Niezpominajek 21, 30-239, Kraków, Poland; n.hordynska@ifr-pan.edu.pl (N.H.); a.maksymowicz@ifr-pan.edu.pl (A.M.); s.grzesiak@ifr-pan.edu.pl (S.G.); 2 Plant Breeding and Acclimation Institute-National Research Institute, 05-870 Błonie, Radzików, Poland

**Keywords:** drought, morphological traits, root system structure, root box—pin board methods, root-basket methods, spring wheat

## Abstract

(1) Background: The study analyzed wheat morphological traits to assess the role of roots structure in the tolerance of drought and to recognize the mechanisms of root structure adjustment to dry soil environment. (2) Methods: Root-box and root-basket methods were applied to maintain an intact root system for analysis. (3) Results: Phenotypic differences among six genotypes with variable drought susceptibility index were found. Under drought, the resistant genotypes lowered their shoot-to-root ratio. Dry matter, number, length, and diameter of nodal and lateral roots were higher in drought-tolerant genotypes than in sensitive ones. The differences in the surface area of the roots were greater in the upper parts of the root system (in the soil layer between 0 and 15 cm) and resulted from the growth of roots of the tolerant plant at an angle of 0–30° and 30–60°. (4) Conclusions: Regulation of root bending in a more downward direction can be important but is not a priority in avoiding drought effects by tolerant plants. If this trait is reduced and accompanied by restricted root development in the upper part of the soil, it becomes a critical factor promoting plant sensitivity to water-limiting conditions.

## 1. Introduction

Roots, as one of the primary organs, play an important role in vascular plants. They are a hidden part of a plant, responsible for growth, development and productivity, anchorage, and supplying stem and leaves with water and nutrients [1,2,3,4,5,6,7,8]. Roots constitute a source of organic matter, they influence the soil structure, aeration, and its biological activity [9,10,11]. Our knowledge on root interaction with soil environment is relatively poor; however, recent advances in imaging and sensor technologies are making root phenomic studies more possible and efficient [2,3].

Root system structure (RSS) is defined as a spatial configuration of roots in the soil profile (refers to the shape and physical space of the roots) and is controlled genetically [12,13,14]. The RSS consists of several components, such as seminal, seminal adventitious, nodal, and lateral roots. The nodal roots build up a framework, while lateral roots provide a network of the root system [15,16,17,18,19]. In cereals, two types of lateral roots can be distinguished according to their length, diameter, and histological structure. The L-type lateral roots are long, thick, and branch into the higher-order lateral roots, while S-type lateral roots are short, slender, and non-branching. Individual components of one root system can differ in origin, age, phenotypic plasticity, anatomical and morphological features, and physiological function [5,16,19,20]. Based on a “root box—pin board” method (also known as “rhizobox”), “concentrated” or “scattered” RSS types were described in cereals [15,18]. “Concentrated” type of the root system develops a greater number of densely distributed nodal roots with a relatively small rooting angle. The second type, designated as “scattered”, develops fewer but longer nodal roots, many of which grow obliquely in the soil profile (larger rooting angle). In “root-basket” method, the RSS is classified as “monomorphic-shallow”, “monomorphic-deep”, and “dimorphic” root systems in cereals [5]. The deep root system effectively absorbs water and nutrients from deeper soil layers [21], while shallow root systems ensure better nutrient uptake from fertilized upper soil profile [4,5]. The dimorphic root system comprises both deep and shallow roots, and thus it can adapt to multiple adverse factors such as water and nutrient shortages [22,23,24].

Soil drought is a common environmental phenomenon affecting plant growth, development, and yield. Strong differences in susceptibility to drought exist among genotypes, varieties, and species, as demonstrated by in various studies in, e.g., wheat [25,26], maize [23,27,28,29], oat [30,31], triticale [24,32,33], grasses [34], *Arabidopsis*, and poplar [35]. Acquisition of water (and mineral nutrients) is primarily determined by the dimension of the entire root zone, root density, their differentiation and elongation, and is closely related to soil water availability [18,33,36,37]. In water-limited environments, plants develop small root systems and the reduction in RSS size is proportional to the changes in water shortage [18]. In cereals exposed to long and severe drought, the rate of root elongation slows down due to reduced nutrient supply to the cortical cell layer. Since soil drought significantly affects the morphology and anatomy of root system components, a symbiosis between the roots and mycorrhizal fungi can be disrupted. As a result, both the flow of carbon from the root system to rhizosphere microorganisms and the flow of oxidized NH_4_ from the microorganisms to the plant could be inhibited [6,15,33]. Further, in the atmosphere, changes of the climatic factors (relative humidity, air temperature) occur more frequently and faster than in the soil, where they are mitigated by greater inertia [5,8,17,28]. Therefore, the relation between the size of the above-ground part and roots (S/R ratio) is of vital importance for the plant water balance. Lower S/R is more advantageous for plant exposed to water-limited environment and is an important mechanism of plant drought tolerance.

The changes in water status of the soil–root–leaf continuum negatively influence various physiological processes, such as intensity of leaf gas exchange and photosynthesis, membrane injury, or chlorophyll content [33,38,39,40]. Plants have developed different mechanisms that can reduce or repair the harmful effects of drought [9,10,25,34,41], including RSS plasticity, which allows plants to adapt to different soil water content [21,41]. Studies published so far have focused mostly on the above-ground organs, and physiological changes in the roots and the relationships between root system architecture and soil water availability are much less understood [29,42,43]. This greatly limits the development of new drought-resistant genotypes in breeding programs. Therefore, the objective of this study was to evaluate changes in root morphological traits in three drought-resistant and three drought-sensitive wheat genotypes grown under long soil drought. The study also aimed at improving methods that would help to determine the root features and their ecological and physiological role. The results of the experiments may help the breeders in determination of genetic diversity of wheat and in the development of new cultivars with improved water use efficiency and water economy.

## 2. Results

### 2.1. Plant Traits Under Control (C) Conditions

**Figure 1:**
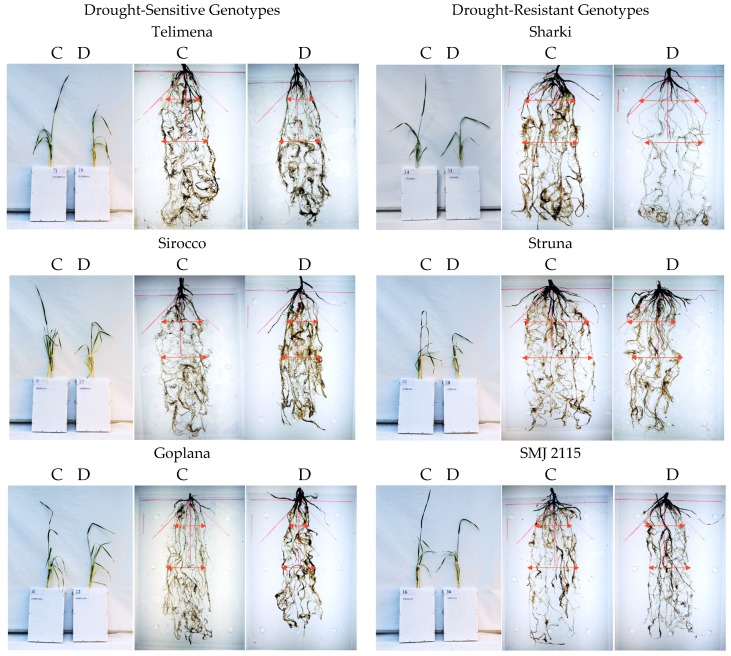
Representative examples of 8-week-old wheat plants and their roots in control (**C**) and drought (**D**) conditions during the root-box experiment. Cvs. “Telimena”, “Sirocco”, and “Goplana” were selected as drought-sensitive genotypes, while cvs. “Sharki”, “Struna”, and “SMJ 2115” as drought-resistant genotypes. The angle of 45° from main root axis is marked in red, and arrows indicate the width of the root system.

**Figure 2:**
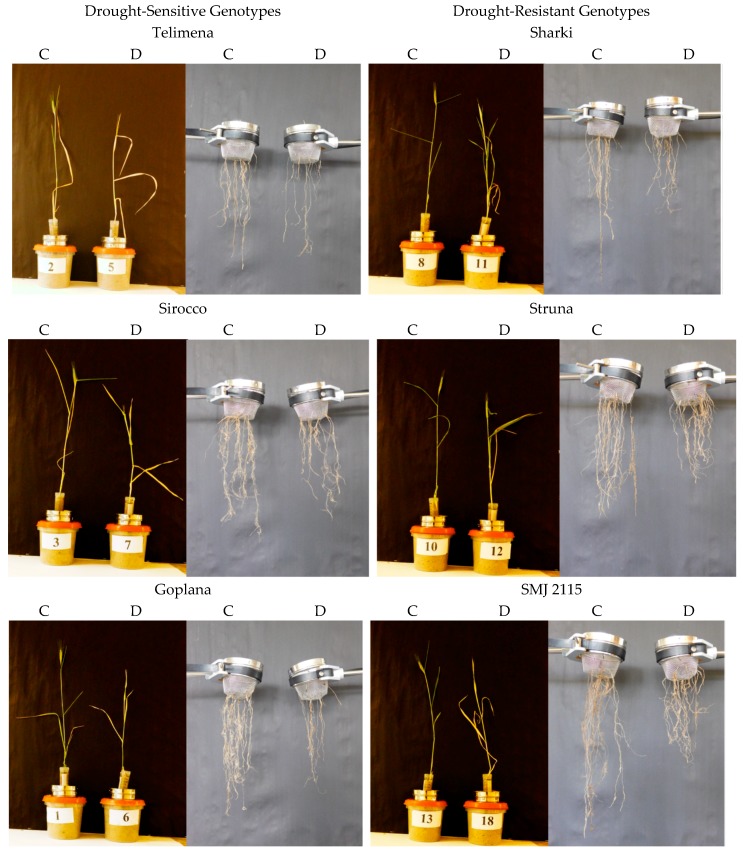
Representative examples of 8-week-old wheat plants and their roots in control (**C**) and drought (**D**) conditions during the root-basket experiment. Cvs. “Telimena”, “Sirocco”, and “Goplana” were selected as drought-sensitive genotypes, while cvs. “Sharki”, “Struna”, and “SMJ 2115” as drought-resistant genotypes.

Wheat genotypes, both sensitive and resistant to drought, developed the “concentrated” and “deep” type of the root system, as most of the roots were located in the range up to 45 from the main axis of the root system (Figure 1 and Figure 2). In control conditions, all genotypes developed one main seminal, two seminal adventitious roots, and a similar number of nodal roots. Root development followed different patterns in the sensitive and resistant genotypes. The drought-resistant genotypes had more extensive and branched root system even at optimal water content.

In root-box experiment under control conditions, most genotypes included in the sensitive group were higher (H) and developed a larger number of leaves (L_No_), when compared to drought-resistant genotypes (Table 1). Dry mass of the above-ground parts (S), the roots (R), and the S/R ratio were also greater. Contrary, total root length (R_L_) and mean root length (R_ML_) were lower in the sensitive than resistant plants (Table 2). The root-basket experiment confirmed higher growth (H) of sensitive plants (Table 3). Dry mass of above-ground parts (S), S + R, and S/R ratio were also greater (Table 3), whereas dry matter (R) and length of nodal roots (R_L_) growing at an angle of 0–30° and 30–60° were smaller in sensitive genotypes than in resistant ones (Table 4).

### 2.2. Plant Traits Under Drought (D vs. C)

The differences between drought-resistant and drought-sensitive genotypes under drought treatment were observed in density of the roots in the upper parts of the root system structure. In the resistant genotypes exposed to drought, a tendency to increase the number of roots in the upper part and occupy space above the angle of 45° was observed (Figure 1 and Figure 2). In root-box containers, drought-sensitive genotypes exhibited significantly decreased height (H), the strongest reduction of the root size, and smaller dry matter of shoot and roots (S, S + R, R_U_, R_B_, R, respectively) when drought conditions were applied (Figure 1, Table 1). The number of sensitive leaves (L_No_) and S/R ratio did not differ significantly in drought-treated and control plants. The sensitive genotypes showed also a significant decrease in nodal root length (R_L_) and mean length of nodal roots (R_ML_) under drought (Figure 1, Table 2). Drought slightly diminished the majority of traits in the drought-resistant genotypes, with an exception of total root dry matter (Figure 1, Table 1), nodal root length (R_L_), and mean length of nodal roots (R_ML_) (Figure 1, Table 2), which were similar in control and drought-treated tolerant genotypes. A total length of two seminal adventitious roots differed among genotypes and it did not depend on plant tolerance to water-limited environment. Lack of statistically significant differences in the length of seminal root were probably due to the duration of the experiment, which was long enough for the roots to finish their growth and reach maximum length. In the root-basket experiment, drought diminished the height of all plants and dry matter of their shoots (Figure 2, Table 3). Importantly, the number of leaves significantly decreased in resistant plants but not in the sensitive ones, while dry mass of roots decreased significantly in sensitive plants but not in resistant ones. As a result, the shoot-to-root ratio dropped significantly only for the resistant genotypes. Furthermore, in drought-sensitive plants growing in root-basket containers, all measured root traits, i.e., root dry matter, number of nodal roots, and length of nodal roots growing at an angle 60–90°, significantly decreased under drought in comparison with control conditions (Table 4). Contrary trends occurred in resistant plants, in which drought increased dry matter of the roots growing at an angle 0–30°, as well as the number of nodal roots and the length of nodal roots growing at an angle 0–60°.

The relative trait change index (RTC) showed that in both root-box and root-basket experiments, the water-limiting conditions caused a greater decrease in H, S, R, and S + R, but not in L_No_ in the sensitive genotypes, when compared to resistant ones (Table 1 and Table 3). Drought-resistant genotypes accumulated greater dry biomass of roots in the upper layer of the soil (at a depth of 0–15 cm) and slightly smaller in the lower layer (at a depth of 15–40 cm) than the sensitive genotypes (Table 1 and Table 3). Roots of the tolerant plants that grew at the angle of 0–60° surpassed the sensitive plant roots in most of the parameters (Table 4). Sensitive genotypes exhibited the highest decrease of nodal root length (Table 2), and diminished all traits of the roots growing at the angle of 60–90°.

For most of the measured traits, significant variations (ANOVA) in the above-ground plant parts and roots were found for six genotypes, two treatments, and genotypes × treatments, but no significant variation in the number of leaves (L_No_) was established (Table 1 and Table 3).

### 2.3. Correlation Between Drought Susceptibility Index (DSI) and Relative Trait Changes (RTC)

Linear correlation coefficients “*r*” between DSI ([26] and Materials and Methods section) and RTC of the measured traits in the plants growing in root-box and root-basket containers were high and statistically significant (Table 5). Contrary to that, insignificant correlation coefficients “r” were calculated for the length of seminal and seminal adventitious roots (0.605 and 0.104) and the number of roots growing at the angle of 0–30° and 30–60° (−0.004 and 0.543).

**Figure 3:**
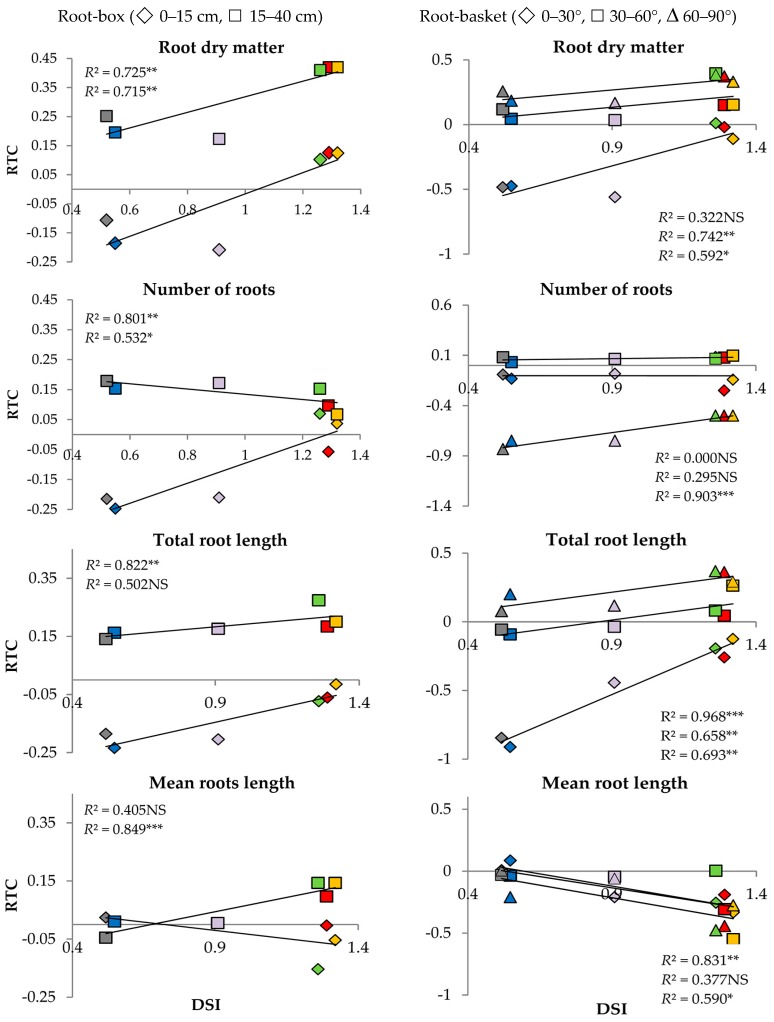
Relationship between drought susceptibility index (DSI) and relative trait change index (RTC) for six wheat genotypes: Telimena (red), Sirocco (yellow), Goplana (green), Struna (blue), Sharki (violet), and SMJ 2115 (grey) in the root-box experiment and the root-basket experiment. NS—regression coefficient (*R*^2^) statistically not significant; *, **, *** *R*^2^ statistically significant at *p* < 0.1, 0.05, and 0.01, respectively.

The relationship between DSI and RTC calculated for the roots is presented in Figure 3. The root-box experiment revealed significant correlations between DSI and root dry matter and between DSI and the number of developed roots in both soil profiles: from 0 to 15 cm and from 15 to 40 cm. Another significant correlation was found for the length of roots growing at 0–15 cm below soil level and mean root length at the depth of 15–40 cm (Figure 3a). The root-basket experiment indicated a significant correlation between DSI and total root length when the roots grew at the angles of 0–30°, 30–60°, and 60–90° (Figure 3b). On the other hand, there was no statistically significant correlation between DSI and root dry matter (R) for the angle of 0–30°, the number of roots for the angle of 0°–30° and 30–60°, and mean root length (R_ML_) for the angle of 30–60°.

### 2.4. Transcriptional Responses to Drought in Root Tissue 

A number of transcripts can show overlapping and specific response under the experimental conditions in drought (Table 6, Supplementary Figures S1–S6). The transcripts up-regulated in roots under drought stress conditions were annotated to genes functional in pathways dependent on the transcription factors (TFs), auxin, cytokinin, mitogen-activated protein kinase, abscisic acid, ethylene, and antioxidative enzymes. Specifically, few transcription factors that make up the RNA polymerase II preinitiation complex (TFIID), a basic leucine zipper (BZIP) TFs, ethylene-responsive TFs, drought-responsive factor-like TFs (DRFL), wax production-like (WXPL) TFs, and further sequence-specific DNA binding TFs like LIM, MYB, MIKC-type MADS-box, NAC, and WRKY were identified to be the main players of drought tolerance via regulation of root traits. Additionally, several genes were found to be regulators of drought responses in wheat via signaling pathways involving an actin filament bundle assembly, transmembrane transport, cell differentiation, cell division, priming of cellular response to stress, and detoxification of reactive oxygen species.

## 3. Discussion

The turn of the 21st century witnessed a drastic decrease in the yield of crops, including rice, wheat, and maize, despite increasing concentrations of CO_2_ that could benefit photosynthesis [44]. Drought turned out to be the most detrimental abiotic stress that limited crop yield by as much as 30–80% [45]. Scientists and breeders are gradually improving crop plant genotypes in terms of drought tolerance using both traditional selection methods and genetic engineering [26,34]. However, tolerance to drought stress is a complex feature, and finding suitable phenotypes requires time-consuming exposure of plants to simultaneously or sequentially occurring stress factors in natural conditions. Further, the progress in breeding is slower than expected and some of the reasons are that the genotypic variation in drought tolerance in crops has not yet been fully comprehended [9,46,47,48], and the genotype itself is not the only factor determining survival in suboptimal conditions [13,14,17,23,33,34,49,50,51]. Our research focused on both aspects in an attempt to recognize: (1) The role of root structure in soil drought tolerance, and (2) the mechanisms of root structure adjustment to the surrounding soil environment in order to increase competitiveness of water uptake by plant roots in dry conditions.

Wheat genotypes used in our study showed a wide range of drought tolerance traits in previous experiments [26]. We found difference and correlation between the physiological traits (photosynthesis, growth of aerial parts, and yield) and drought susceptibility index (DSI), proving the effect of genetic variation on the degree of drought tolerance, and grounds for the selection of suitable genotypes for water-limited environments. In the present study, the traits of the above-ground plant parts (height, leaf number, shoot dry matter) correspond to those for roots (dry matter, number, length), confirming that drought conditions were correctly optimized and applied in our experimental system. Shoot height and dry matter diminished severely in sensitive plants and moderately in resistant ones. Surprisingly, leaf number remained similar in sensitive genotypes but lowered significantly in resistant ones. Some leaves of sensitive plants showed slight chlorotic spots. Visible symptoms of drought resulted from a sequence of physiological processes, i.e., stomatal closure, reduced tissue turgor, leaf gas exchange, limited supply of CO_2_ to the mesophyll cells, and, subsequently, a decrease in photosynthesis rate [20,26,34,49,50]. The ability to maintain the structure and function of photosynthetic membranes under water deficit is one of the most important tolerance mechanisms. Efficient mechanisms that protect the function and structure of the membranes by stronger binding of molecules and maintaining intact lipid–protein complexes can prevent, i.e., disruption of thylakoid membranes and chlorophyll antennas [26,34,38,41,50,52]. Although smaller, the resistant leaves did not show the visible symptoms of damaged photosynthetic apparatus, thus confirming better acclimation to drought stress.

Root distribution in individual genotypes depended also on water availability and individual tolerance to drought. Water-limiting conditions exacerbated the decrease of the root system dry matter and reduced the number, length, and diameter of nodal and lateral roots in drought-sensitive genotypes, similarly as in many other studies [4,13,17,18,21,38,53,54]. Drought-resistant genotypes featured improved RSS (more extensive and branched root system), even at optimal water content. The most interesting and important differences between sensitive and tolerant plants included: (1) Better adaptation to reduced soil water content; and (2) greater plasticity of morphological changes in the root architecture. In the first case, the number of leaves decreased and the root system showed more intense development in the resistant plants exposed to drought. In the sensitive plants, number of leaves did not differ in control and water-limiting conditions, but dry mass of roots declined significantly. As a result, resistant genotypes had a lower S/R ratio. Lowering the number of leaves in dry conditions is a physiological priority that enables reduction of transpiration and improvement of plant water management and water use efficiency. Consequently, grain yield is greatly reduced; however, it remains sufficient to produce succeeding generations (Darwinian fitness). On the other hand, plants maintain more favorable distribution and supply of photosynthesis products to below-ground parts that promoted root growth and development [10,12,14,36,46,55]. Increased root surface area can improve nutrient and water absorption and contribute to the final yield and development of drought tolerance-related traits [44]. Higher number of roots can also protect against lodging and breaking roots in drying soil. In the second case, an intermediate number of roots seems to provide superior plant performance, as too extensive root system development may result in competition for soil and metabolic resources [11,53,56].

In our research, the differences in the root development between tolerant and sensitive genotypes were particularly visible in the upper part of the root system during soil drought. This resulted from a more vigorous growth of tolerant roots in the soil layer at the depth of 0–15 cm. More dry matter accumulated close to the ground level as a consequence of the highest number of nodal roots and their higher length. In the tolerant genotypes exposed to drought, growth and development of the root system were executed mainly by the roots growing at the angle of 0–30° and 30–60°. In the sensitive genotypes and in control conditions, dry matter, number of nodal roots, and their length close to the ground level were much smaller. In the plants sensitive to drought, all measured traits for the roots growing at the angle of 60–90° also decreased significantly under drought. These differences suggest that the roots of resistant genotypes grew through the soil more extensively than the roots of sensitive genotypes. The mechanism of plant root biomass allocation in the soil space translates into specific physiological functioning. Fast growth results in a deeper root system, formation of a denser root network in the upper soil layer, and in turn increases exploitation of soil resources. Higher root density in the active root zone area (up to 30 cm) improves water and nutrient uptake and enhances yield of the plants grown under water deficit conditions [57]. Wider root angles could also reduce the energy inputs to penetrate deeper soil layers to access water during limited rainfall [37] and reduce surface soil evaporative losses. Contrary to that, a more vertical angle and a greater number of seminal roots in wheat plants have been associated with more compact and deeper roots. As sufficient moisture remains available in deeper soil layers under drought, one can expect that deep roots are essential for small-statured crops, such as wheat, to extract water. However, wheat genotypes with deeper roots, higher root density in deeper soil layers, and smaller root density at the surface produce higher yield under rain-fed conditions [58].

Drought stress tolerance as a polygenic trait involves the expression of many sets of genes governing morpho-physiological traits, including root architecture [59,60,61]. The meta-analytical approach and analysis of multiple transcriptomic data have uncovered many transcription regulators and key genes. They can work via stress signaling, stress transduction, biosynthesis of stress-related proteins, enzymes and metabolites to coordinate the drought stress responses of wheat roots. We identified transcription factors such as TFIID, BZIP TFs, ethylene-responsive TFs, DRFL, WXPL TFs, LIM, MYB, MIKC-type MADS-box, NAC, and WRKY, which can be the enhancers and positive regulators of root traits under drought. For example, TFs of the MYB can be associated with the majority of biochemical and physiological processes in plants, via regulation of primary and secondary metabolism, hormone synthesis (ABA, ethylene, auxin), and signaling [62,63,64]. In our multi-transcriptome analysis of roots exposed to water deficit, numerous up-regulated MYB-encoding genes were found. It was reported that MYB can be involved in lateral root formation and in the biosynthesis of phenylpropanoid compounds, such as lignin [64]. The lower lignin content in roots can promote cell elongation of roots growing under drought; on the other hand, a higher rate of lignin accumulation can be required under prolonged drought to increase the mechanical strength of developed root tissues. Therefore, they can play a key role in root development and root structure arrangement. Similar to MYB TFs, abundant NAC TFs were up-regulated in drought-exposed roots. They can regulate development of thicker roots with enlarged stele, cortex, and epidermis, control a higher number of cells in stele and aerenchyma tissues, and induce lateral roots development and greater root biomass [64,65,66]. In our meta-analysis of multiple transcriptomes, NAC expression was not limited to the root; although, some of the NACs were found specifically or preferentially in this organ. Further, a direct role of ABA-dependent WRKY transcription factors was evident from their up-regulation in response to drought stress. The role of WRKY as a regulator of reactive oxygen species and antioxidant accumulation, cellular membrane stability, and altered osmotic adjustment was suggested [59,62,66]. We found, that the important mechanisms of drought sensing by roots were also regulated by hormones. The predominant role was attributed to abscisic acid (ABA), ethylene, auxins, and cytokinins. They are key regulators of complex heat–drought stress genetic networks in wheat [59,61,62,66]. ABA is important for early response to drought stress, but its high content in roots is not beneficial under prolonged and severe stress. Although the elevated expression of the zeaxanthin epoxidase (ZEP) encoding genes was not found in our transcriptomic analysis in roots, the other ABA-related genes such as genes encoding HVA22-like proteins were up-regulated. ABA biosynthesis is correlated with auxin transport to the root tips, which, in turn, enhances proton secretion in this zone [64]; therefore, their coexpression would be beneficial for development and morphology of the roots, as suggested by meta-analysis. Our analysis also indicates that universal stress players like antioxidative enzymes or mitogen-activated protein kinase play an important role in the roots during drought. We showed that their expression in wheat can be organ-specific. Particularly, two mitogen-activated protein kinases, as well as superoxide dismutase, catalase, glutathione reductase, and flavin-containing monooxygenase, can play an important role in acclimation of the root system to drought conditions. The above study of transcriptome-wide variation suggests the genes, which provide a promising example of multiple roles in drought stress, can be targets of molecular breeding strategies for improving drought tolerance, or at least candidate genes and putative molecular markers.

Concluding, a wide-spreading and much-branched root system is a specific feature of tolerant plants. Such a type of root system is required for stress avoidance or reduction of drought effects in a more efficient way than the deep-type root system. Extended root architecture and root size, also in the upper soil layer, can provide plants with a competitive edge regarding water uptake. Therefore, wide distribution of the roots in soil greatly affects the final crop yield. In breeding programs, selection of plants with both branched and deep roots should be taken into account to enhance soil water capture by new varieties and to help in yield stabilization under water stress conditions. Root traits such as specific root length, specific root area, and root density should be considered with the wide root angle, as they comprise the key traits for improving plant productivity under drought. Regulation of cell elongation at the root tip to bending of the root in a more downward direction can be important, but is not a priority in avoiding drought effects by tolerant wheat plants. However, if this trait is reduced and accompanied by restricted root development in the upper part of the soil, it becomes a critical factor promoting plant sensitivity to water-limiting conditions.

## 4. Materials and Methods

Plant material. Six commercial and breeding forms of wheat from DANKO Plant Breeders Ltd and Plant Breeding Centre Smolice Ltd., Co, were choosen based on our previous study. We selected three drought-sensitive (Sirocco, Telimena, Goplana) and three drought-resistant (Sharki, Struna, SMJ 2115) genotypes. Their drought susceptibility index (DSI = (1 − D/C)/(1 − xD/xC); where C, D—dry matter of aboveground part of the plant in control (C) and drought (D) conditions, xD, xC—average values for all examined genotypes) was 1.32, 1.29, 1.26, 0.91, 0.55, and 0.52, respectively [26], which gives clustering of sensitive and tolerant groups, but also more or less linear genotypic dependency on tolerance. The selected genotypes had also different origin, thus different genotypic diversity.

**Figure 4:**
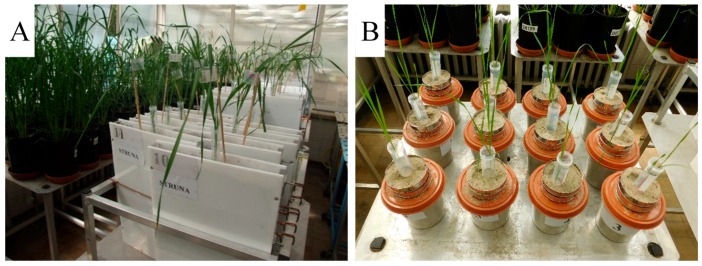
Wheat plants in root-box (**A**) and root-basket (**B**) containers.

Growth conditions. The experiment was carried out in an air-conditioned glasshouse (Kraków, Poland), under the following day/night conditions: Temperature 23/18 ± 2.5 °C photoperiod 14/10, and relative humidity 70% ± 5%. Photosynthetically active radiation (PAR) was set to 350 ± 20 μmol photons m^−2^ s^−1^. Plants were grown in custom-made root-box or root-basket containers (Figure 4, Supplementary Figures S7 and S8) filled with a mixture of universal potting soil (Holas, Poland) and quartz-sand (Chemoform AG, Germany) in proportion 1:2 (*v*/*v*). Soil was mixed with compound fertilizer at the rate N: 2.8 mg, P: 1.8 mg, K: 1.4 mg per 1 kg of the soil substrate. Air-dried soil substrate was sieved through a 0.25 cm mesh and soil substrate compaction in root containers was set to 1.3 g cm^−3^. Field soil water capacity (FWC) for the soil mixture was determined according to the Kopecky method. Briefly, air-dried soil samples (100 cm^3^) were placed inside metal cylinders with 1 mm holes at the bottom. The cylinders were then placed inside a container filled with water for 30 min. The maximal soil water content in the samples was 0.43 g cm^−3^ after 8 h, and it decreased to 0.21 g cm^−3^ after 48 h. This final value was assumed as 100% FWC [67].

The “root box–pin board” and “root-basket“ methods were applied to enable non-destructive cleaning and analysis of all intact compartments of the root system [15]. The “root box–pin board method” utilized a plexiglass root-box (height 40 cm, width 20 cm, thickness 2 cm), a pin-board for sampling the roots, and a perforated polyethylene sheet (envelope) for handling and preserving the roots. The “root-basket” included two baskets with a horizontal mesh (diameter 9.0 cm, holes 0.25 × 0.25 cm, HTL-Trading B.V., Nederland), the upper one 2.0 cm high, and the lower one 6.0 cm high to maintain a constant distance between the grain placed in the upper basket and the mesh of the lower basket. The set of root baskets was placed in a pot (diameter 12.0 cm, height 13.0 cm). The root-box and root-basket containers were weighed three times per week and the amount of water lost through evapotranspiration was refilled to keep the constant mass.

In each root-box or root-basket container, a single pregerminated grain was planted at a depth of 2 cm (March 30, 2019). At the harvest (27 May 2019), the shoots and roots were separated. Roots were sampled in an envelope placed on the pin-board by removing the soil substrate with a gentle water stream. Root samples were preserved in formalin–acetic acid–ethanol (FAA) solution fixative (Fisher Scientific) and scanned.

Drought stress. Soil drought was induced by limiting watering. Drought treatment (D) at the level of 30–35% FWC was maintained for 6 weeks (from 14 to 56 days of plant growth). For control (C) treatment, soil water content was maintained at 65–70% FWC from sowing to harvest. The water content in pots was controlled during whole experiment by weighting of the containers. The loss of water caused by transpiration and evaporation was refilled every 2–3 days.

Plant traits. At the harvest, the above-ground parameters, such as plant height (H), shoot dry matter (S), and the number of leaves (L_No_), were assessed. The below-ground traits were recorded as a number (R_No_) and length (R_L_) of particular components of the root system (seminal root, seminal adventitious root, nodal root). In the root-box experiment, dry matter of roots (R) was analyzed for two levels of soil profile, i.e., roots collected from the layer of 0–15 cm below ground (RU), and from 15 to 40 cm (RB). The root-basket experiment assessed the number and length of roots growing at the angles of 0–30°, 30–60°, and 60–90° (horizontal ground line = 0°; vertical main root axis = 90°). Dry matter of the above-ground plant parts (S) and roots (R) was measured after drying at 65 °C for 72 h. For each genotype and treatment, three plants from root-boxes and four plants from root-basket containers were collected.

The results are presented as relative trait change index RTC = (C−D)/C, where C represents control, and D refers to drought treatment. Positive values of RTC indicate a decrease of the trait value in comparison with control plants, and negative values of RTC indicate an increase of the trait value vs. control plants.

Meta-analysis of transcripts. The meta-analytical approach of Genevestigator (Genevestigator, Plant Biology, https://www.genevestigator.ethz.ch/, accessed on 27 October 2019) was used to study transcriptomes related to transcription factors, auxin, cytokinin, gibberellin, mitogen-activated protein kinase, abscisic acid, ethylene, and antioxidative enzymes. Multiple RNAseq experiments were chosen on the mRNA-Seq Gene Level *Triticum aestivum* platform. The experimental conditions were defined by the selection of drought and dehydration perturbations from the experiments with accession number TA-00015, TA-00025, TA-00028, TA-00030, TA-00042, TA-00045, TA-00059, TA-00073, TA-00128, TA-00138, and TA-00139. A filter “Anatomy” was used to analyze the transcriptomic data in order to gene expression in different plant organs, including roots. The Similarity Search Tool was used to define the Euclidean distance with the optimal leaf-ordering for both transcripts and wheat anatomy. All data are presented as heatmaps, which represent the maximum level of expression for a given probe across all measurements available in the database for this probe (Supplementary Figures S1–S6). The transcripts with the highest expression were selected and described (Table 6). A functional information and gene ontology were assigned using the EnsemblPlants (EnsemblPlants, https://plants.ensembl.org/index.html, accessed on 27 October 2019) and UniProt (UniProt, https://www.uniprot.org/, accessed on 27 October 2019) databases.

Statistical analysis. Statistical significance of data in the ANOVA analysis was evaluated in STATISTICA 12.0 (Statsoft, Tulsa, OK, USA). Three biological replicates were used in each root-box experiment, and four biological replicates were used in each root-basket experiment per plant and treatment. The means were compared using Duncan’s multiple range test at 0.05 level of probability. We also calculated the F-values, linear correlation coefficients, and probability levels.

## 5. Conclusions

Our study contributed to the understanding of RSS responses to soil drought and determining root morphological traits of wheat genotypes with different drought susceptibility. The results suggest that drought triggers specific modifications of the wheat root system structure that involve changes in the roots biomass, size, and distribution at different levels of the soil profile and changes in the angle of root growth in relation to the main root axis. The methods being developed are useful tools to capture and analyze the complex root systems in laboratory conditions under a limited space, but on the other hand, the results correspond to a high degree to (previously published) field phenotyping.

## Figures and Tables

**Table 1:** Effect of soil drought on wheat genotypes differing in their drought susceptibility index (DSI) during the root-box experiment. Plant height (H); leaf number (L_No_); dry matter of shoot (S); dry matter of roots growing in two soil levels (R_U_, upper from 0 to 15 cm; R_B_, bottom from 15 to 40 cm); total root dry matter (R); total plant dry matter (S + R); shoot-to-root ratio (S/R); control treatment (C); drought treatment (D); relative trait change index (RTC); the mean of plant group is provided together with the standard error (SE); (*n* = 3).

Genotype	Treatment	H (cm)	L_No_	Dry Matter (g plant^−1^)					
				S	R_U_	R_B_	R (R_U_ + R_B_)	S + R	S/R
**Drought-Sensitive Genotypes (DSI > 1.0)**									
Telimena	C	51.30 b	6.70 a	1.48 a	0.28 b	0.55 a	0.82 ab	2.30 a	1.80 a
	D	40.00 de	6.30 ab	0.95 d	0.24 c	0.32 e	0.56 b	1.51 c	1.70 ab
	RTC	0.22	0.06	0.36	0.14	0.42	0.32	0.34	0.06
Sirocco	C	58.00 a	6.30 ab	1.44 ab	0.28 b	0.55 a	0.83 a	2.27 a	1.74 a
	D	44.00 d	6.00 b	0.97 d	0.24 c	0.32 e	0.56 b	1.53 c	1.73 a
	RTC	0.24	0.05	0.33	0.14	0.42	0.32	0.33	0.01
Goplana	C	51.70 b	6.30 ab	1.33 b	0.25 bc	0.51 ab	0.84 a	2.17 a	1.59 cd
	D	41.00 de	5.70 bc	0.79 e	0.23 c	0.30 e	0.53 d	1.32 d	1.49 d
	RTC	0.21	0.10	0.41	0.08	0.41	0.37	0.39	0.06
Mean ± SE	C	53.70 ± 2.17	6.40 ± 0.10	1.41 ± 0.05	0.27 ± 0.01	0.54 ± 0.01	0.83 ± 0.01	2.24 ± 0.04	1.71 ± 0.06
	D	41.70 ± 1.20	6.00 ± 0.20	0.90 ± 0.05	0.24 ± 0.05	0.31 ± 0.01	0.55 ± 0.01	1.45 ± 0.07	1.64 ± 0.08
	RTC	0.22	0.07	0.36	0.11	0.43	0.34	0.35	0.04
**Drought-Resistant Genotypes (DSI < 1.0)**									
Sharki	C	46.30 c	6.00 b	1.34 b	0.28 b	0.56 a	0.82 a	2.18 a	1.59 cd
	D	38.00 de	4.70 d	1.10 cd	0.34 a	0.47 c	0.81 ab	1.91 b	1.37 e
	RTC	0.18	0.22	0.18	−0.21	0.16	0.01	0.12	0.14
Struna	C	47.30 c	6.00 b	1.37 b	0.27 bc	0.53 ab	0.80 b	2.17 a	1.71 ab
	D	38.70 de	4.70 d	1.10 c	0.32 a	0.43 cd	0.75 bc	1.85 b	1.49 d
	RTC	0.18	0.22	0.20	−0.18	0.19	0.06	0.15	0.13
SMJ 2115	C	49.70 bc	6.30 ab	1.33 b	0.24 c	0.54 ab	0.81 ab	2.14 ab	1.65 bc
	D	41.30 de	5.30 c	0.99 d	0.30 ab	0.40 d	0.73 c	1.72 c	1.36 e
	RTC	0.17	0.16	0.26	−0.25	0.25	0.10	0.20	0.18
Mean ± SE	C	47.80 ± 1.00	6.10 ± 0.10	1.35 ± 0.01	0.27 ± 0.00	0.54 ± 0.01	0.82 ± 0.01	2.16 ± 0.01	1.65 ± 0.03
	D	39.30 ± 1.00	4.90 ± 0.20	1.06 ± 0.04	0.32 ± 0.01	0.43 ± 0.02	0.76 ± 0.02	1.82 ± 0.06	1.40 ± 0.04
	RTC	0.18	0.20	0.21	−0.19	0.20	0.07	0.16	0.15
**ANOVA**									
**Variable**	**df**	**H**	**L_No_**	**S**	**R_U_**	**R_B_**	**R**	**R + S**	**S/R**
Genotype (G)	5	**	ns	*	**	**	*	**	***
Treatment (T)	1	*	ns	*	*	*	*	*	*
G × T	5	*	ns	*	*	*	*	*	*

Different letters indicate significant differences within columns, according to the Duncan’s test (*p* < 0.05); *, **, ***—statistical significance at *p* < 0.1, 0.05, 0.01, respectively; ns—no significant differences; df—number of freedom degree. Based on RTC, the genotypes were divided into three groups: yellow—statistically significant decrease of traits under D in comparison with C; green—statistically significant increase of trait under D in comparison with C; blue—no statistically significant differences between C and D.

**Table 2:** Effect of soil drought on wheat genotypes differing in their drought susceptibility index (DSI) during the root-box experiment. Length of one seminal root (R1S_L_); total length of two seminal adventitious roots (R2SA_L_); number of nodal roots (R_No_); length of nodal roots (R_L_); mean root length (R_ML_); control treatment (C); drought treatment (D); relative trait change index (RTC); the mean of plant group is provided together with the standard error (SE); (*n* = 3).

Genotype	Treatment	Seminal Roots	Seminal Adventitious Roots	Nodal Roots		
		R1S_L_ (cm)	R2SA_L_ (cm)	R_No_	R_L_ (cm)	R_ML_ (cm)
**Drought-Sensitive Genotypes (DSI > 1.0)**						
Telimena	C	43.2 a	43.2 c	16.7 a	243.7 d	14.6 d
	D	40.2 a	49.8 a	16.0 ab	223.3 e	14.0 d
	RTC	0.07	−0.15	0.04	0.08	0.04
Sirocco	C	44.3 a	41.8 d	16.7 a	262.2 c	16.0 c
	D	42.0 a	45.7 b	15.7 ab	230.7 de	14.7 d
	RTC	0.05	−0.09	0.06	0.12	0.08
Goplana	C	41.7 a	50.7 a	16.3 a	279.8 a	17.3 b
	D	41.6 a	43.5 c	15.3 b	236.0 d	15.4 cd
	RTC	0.00	0.14	0.06	0.16	0.11
Mean ± SE	C	42.2 ± 0.7	42.4 ± 1.4	16.6 ± 0.1	261.9 ± 10.4	16.1 ± 0.7
	D	41.1 ± 0.8	39.4 ± 1.7	15.7 ± 0.2	230.0 ± 3.7	14.7 ± 0.4
	RTC	0.03	0.07	0.05	0.12	0.09
**Drought-Resistant Genotypes (DSI < 1.0)**						
Sharki	C	42.3 a	44.3 bc	15.3 b	265.2 c	17.3 b
	D	41.0 a	38.5 f	15.0 b	257.3 c	17.2 b
	RTC	0.03	0.13	0.02	0.03	0.01
Struna	C	43.0 a	37.8 f	16.0 a	297.7 a	18.6 a
	D	42.7 a	46.7 b	15.3 b	283.3 b	18.4 ab
	RTC	0.01	−0.23	0.04	0.05	0.01
SMJ 2115	C	41.0 a	32.3 g	16.0 a	283.5 b	17.7 ab
	D	43.3 a	40.5 ef	16.0 a	275.0 bc	17.2 b
	RTC	−0.06	−0.25	0.00	0.03	0.03
Mean ± SE	C	42.6 ± 0.5	40.0 ± 2.2	15.8 ± 0.2	282.1 ± 9.4	17.9 ± 0.4
	D	41.9 ± 0.6	38.6 ± 1.9	15.4 ± 0.3	271.8 ± 7.6	17.7 ± 0.4
	RTC	0.02	0.04	0.03	0.04	0.01
**ANOVA**						
**Variable**	**df**	**R1S_L_**	**R2SA_L_**	**R_No_**	**R_L_**	**R_ML_**
Genotype (G)	5	ns	*	ns	*	ns
Treatment (T)	1	ns	ns	ns	*	*
G × T	5	ns	ns	ns	ns	*

Different letters indicate significant differences within columns, according to the Duncan’s test (*p* < 0.05); *—statistical significance at *p* < 0.1; ns—no significant differences; df—number of freedom degree. Based on RTC, the genotypes were divided into three groups: yellow—statistically significant decrease of traits under D in comparison with C, green—statistically significant increase of trait under D in comparison with C, blue—no statistically significant differences between C and D.

**Table 3:** Effect of soil drought on wheat genotypes differing in their drought susceptibility index (DSI) during the root-basket experiment. Plant height (H); leaf number (L_No_); dry matter of shoot (S); total root dry matter (R); total plant dry matter (S + R ); shoot-to-root ratio (S/R); control treatment (C); drought treatment (D); relative trait change index (RTC); the mean of plant group is provided together with the standard error (SE); (*n* = 4).

Genotype	Treatment	H (cm)	L_No_	Dry Matter (g plant^−1^)			
				S	R	S + R	S/R
**Drought-Sensitive Genotypes (DSI > 1.0)**							
Telimena	C	46.50 b	5.50 a	1.67 a	0.97 a	2.64 a	1.72 b
	D	35.00 ef	5.20 ab	1.07 de	0.66 d	1.73 f	1.62 b
	RTC	0.25	0.05	0.36	0.32	0.34	0.05
Sirocco	C	46.70 b	5.20 ab	1.62 a	0.96 ab	2.58 ab	1.69 b
	D	39.00 ef	5.00 bc	1.10 d	0.64 d	1.74 f	1.72 b
	RTC	0.16	0.04	0.32	0.33	0.33	−0.25
Goplana	C	53.00 a	5.20 ab	1.63 a	0.88 abc	2.51 abc	1.85 a
	D	35.90 ef	5.00 bc	0.97 e	0.64 d	1.61 f	1.52 d
	RTC	0.32	0.04	0.40	0.27	0.36	−0.14
Mean ± SE	C	48.70 ± 2.10	5.30 ± 0.10	1.64 ± 0.02	0.94 ± 0.03	2.58 ± 0.04	1.75 ± 0.05
	D	36.60 ± 1.20	5.10 ± 0.10	1.05 ± 0.04	0.63 ± 0.02	1.68 ± 0.05	1.67 ± 0.03
	RTC	0.25	0.04	0.36	0.32	0.35	0.05
**Drought-Resistant Genotypes (DSI < 1.0)**							
Sharki	C	42.2 cd	5.2 ab	1.50 b	0.93 ab	2.43 bc	1.61 c
	D	34.2 f	4.0 e	1.23 c	0.85 bc	2.08 d	1.45 e
	RTC	0.19	0.23	0.18	0.09	0.14	0.10
Struna	C	41.5 cd	5.0 bc	1.49 b	0.97 a	2.47 bc	1.54 d
	D	33.0 f	4.5 d	1.23 c	0.91 abc	2.14 d	1.35 f
	RTC	0.20	0.10	0.17	0.06	0.13	0.12
SMJ 2115	C	44.7 bc	5.5 a	1.49 b	0.94 ab	2.43 c	1.59 c
	D	36.3 ef	4.7 cd	1.11 d	0.80 b	1.91 e	1.39 f
	RTC	0.19	0.15	0.26	0.15	0.21	0.12
Mean ± SE	C	42.80 ± 1.00	5.20 ± 0.20	1.49 ± 0.00	0.95 ± 0.01	2.44 ± 0.01	1.58 ± 0.02
	D	34.50 ± 1.00	4.40 ± 0.20	1.19 ± 0.04	0.85 ± 0.03	2.04 ± 0.07	1.40 ± 0.03
	RTC	0.20	0.16	0.20	0.10	0.16	0.11
**ANOVA**							
**Variable**	**df**	**H**	**L_No_**	**S**	**R**	**S + R**	**S/R**
Genotype (G)	5	ns	*	**	**	**	***
Treatment (T)	1	*	ns	*	*	*	*
G × T	5	ns	ns	*	*	*	**

Different letters indicate significant differences within columns, according to the Duncan’s test (*p* < 0.05); *, **, ***—statistical significance at *p* < 0.1, 0.05, 0.01, respectively; ns—no significant differences; df—number of freedom degree. Based on RTC, the genotypes were divided into three groups: yellow—statistically significant decrease of traits under D in comparison with C, green—statistically significant increase of trait under D in comparison with C, blue—no statistically significant differences between C and D.

**Table 4:** Effect of soil drought on wheat genotypes differing in their drought susceptibility index (DSI) during the root-basket experiment. Total root dry matter (R); number of nodal roots (R_No_); length of nodal roots (R_L_); control treatment (C); drought treatment (D); relative trait change index (RTC); the mean of plant group is provided together with the standard error (SE); (*n* = 4).

**Genotype**	**Treatment**	**R**			**RNo**			**RL (cm)**		
		**0–30°**	**30–60°**	**60–90°**	**0–30°**	**30–60°**	**60–90°**	**0–30°**	**30–60°**	**60–90°**
**Drought-Sensitive Genotypes (DSI > 1.0)**										
Telimena	C	0.04 g	0.16 cd	0.77 a	0.25 g	2.00 f	9.75 a	1.1 de	40.6 e	185.0 a
	D	0.04 g	0.13 d	0.49 c	0.30 g	2.50 e	9.00 b	1.2 de	38.1 f	105.6 f
	RTC	0.00	0.19	0.36	−0.20	−0.25	0.08	−0.09	0.06	0.43
Sirocco	C	0.04 g	0.15 d	0.68 b	0.50 fg	1.75 f	7.75 de	3.0 de	50.3 c	141.8 d
	D	0.05 fg	0.13 d	0.46 cd	0.75 g	2.00 f	7.00 f	3.4 de	33.2 g	90.1 g
	RTC	−0.25	0.13	0.32	−0.50	−0.14	0.10	−0.13	0.34	0.36
Goplana	C	0.04 g	0.26 a	0.66 b	0.50 fg	3.00 c	7.50 e	5.4 d	40.6 e	98.5 fg
	D	0.04 g	0.16 cd	0.44 cd	0.75 g	2.75 de	7.00 f	4.5 de	45.6 d	130.0 d
	RTC	0.00	0.40	0.33	−0.50	0.08	0.07	0.16	−0.12	−0.32
Mean ± SE	C	0.04 ± 0.00	0.18 ± 0.4	0.71 ± 0.07	0.43 ± 0.07	2.25 ± 0.38	8.33 ± 0.08	3.2 ± 0.7	43.8 ± 0.1	141.8 ± 0.3
	D	0.04 ± 0.01	0.14 ± 0.1	0.46 ± 0.02	0.60 ± 0.15	2.42 ± 0.22	8.08 ± 0.46	3.0 ± 0.6	39.0 ± 0.2	108.6 ± 0.2
	RTC	0.09	0.22	0.35	−0.40	−0.08	0.03	0.06	0.11	0.23
**Drought-Resistant Genotypes (DSI < 1.0)**										
Sharki	C	0.07 ef	0.27 a	0.58 bc	1.00 d	3.75 b	8.00 cd	6.5 d	57.1 b	155.6 bc
	D	0.11 bc	0.26 a	0.48 cd	1.75 b	4.25 a	8.00 cd	11.8 bc	63.8 a	117.5 e
	RTC	−0.57	0.04	0.17	−0.75	−0.13	0.00	−0.81	−0.12	0.24
Struna	C	0.08 de	0.25 ab	0.64 b	1.00 d	3.00 c	7.75 de	13.5 b	61.3 a	157.0 b
	D	0.13 ab	0.24 b	0.54 c	1.75 b	3.75 b	8.00 cd	19.5 a	64.0 a	134.4 d
	RTC	−0.63	0.04	0.16	−0.75	−0.25	−0.03	−0.44	−0.04	0.14
SMJ 2115	C	0.10 cd	0.19 c	0.64 b	1.50 c	2.75 de	9.25 a	11.0 c	50.0 c	169.1 b
	D	0.15 a	0.17 c	0.48 cd	2.75 a	3.00 c	7.50 e	15.0 b	53.0 c	153.3 c
	RTC	−0.48	0.11	0.25	−0.83	−0.09	0.19	−0.36	−0.06	0.09
Mean ± SE	C	0.08 ± 0.01	0.24 ± 0.02	0.62 ± 0.02	1.17 ± 0.17	3.17 ± 0.30	8.33 ± 0.46	10.30 ± 0.30	56.10 ± 0.10	160.60 ± 0.10
	D	0.13 ± 0.01	0.22 ± 0.03	0.50 ± 0.02	2.08 ± 0.33	3.67 ± 0.36	7.83 ± 0.17	15.40 ± 0.03	60.30 ± 0.10	135.00 ± 0.10
	RTC	−0.63	0.08	0.19	−0.78	−0.16	0.06	−0.49	−0.07	0.16
**ANOVA**										
**Variable**	**df**	**R**			**RNo**			**RL (cm)**		
		**0–30°**	**30–60°**	**60–90°**	**0–30°**	**30–60°**	**60–90°**	**0–30°**	**30–60°**	**60–90°**
Genotype (G)	5	**	*	*	***	**	*	**	**	**
Treatment (T)	1	*	*	*	*	ns	ns	**	*	*
G × T	5	*	*	*	**	ns	ns	ns	ns	*

Different letters indicate significant differences within columns, according to the Duncan’s test (*p* < 0.05); *, **, ***—statistical significance at *p* < 0.1, 0.05, 0.01, respectively; ns—no significant differences; df—number of freedom degree. Based on RTC, the genotypes were divided into three groups: yellow—statistically significant decrease of traits under D in comparison with C, green—statistically significant increase of trait under D in comparison with C, blue—no statistically significant differences between C and D.

**Table 5:** Correlation coefficient “*r*” between selected traits and relative trait change index (RTC) for wheat genotypes grown in root-box and root-basket containers. Plant height (H); leaf number (L_No_); dry matter of shoot (S); total root dry matter (R); total plant dry matter (S+R); shoot-to-root ratio (S/R); number of nodal roots (R_No_); length of nodal roots (R_L_); mean root length (R_ML_); (df = 4).

Container	H	L_No_	Dry Matter				Root Traits		
			S	R	S + R	S/R	R_No_	R_L_	R_ML_
Root-box	0.881 **	−0.803 **	0.751 *	0.876 **	0.818 **	−0.908 **	0.899 **	0.859 **	0.733 *
Root-basket	0.943 ***	−0.918 ***	0.771 *	0.818 **	0.794 *	−0.779 *	0.918 **	.0.906 **	0.843 **

*, **, ***—statistical significance at *p* < 0.1, 0.05, 0.01, respectively.

**Table 6:** Selected transcripts up-regulated in response of the roots to water deprivation. Analysis of multiple RNAseq experiments with meta-analytical approach of Genevestigator (details in Supplementary Figures S1–S6).

Ensembl Gene/Gene ID	UniProtKB Function/Process
**Transcription Factors**	
Transcription initiation factor TFIID subunit 10/ TraesCS7B02G097300	DNA-templated transcription, mediating promoter responses to various activators and repressors
Ethylene responsive transcription factor 5a/ TraesCS5B02G214400	
BZIP transcription factor B/TraesCS6B02G364000	DNA binding, ABA signaling
DRF-like transcription factor DRFL2a/TraesCS6B02G331000	DNA binding, transcription regulation
DRF-like transcription factor DRFL2b/TraesCS6D02G281200	
DRF-like transcription factor DRFL2c/TraesCS6A02G301900	
Drought-responsive factor-like transcription factor DRFL1a/TraesCS5D02G200900	
WXPL1B transcription factor/TraesCS5B02G193200	
R2R3-MYB transcription factor TaMyb1D/TraesCS5D02G335700	DNA binding, cell differentiation
Transcription factor LIM/TraesCS5D02G115000	Actin filament binding, metal ion binding, mRNA binding, actin filament bundle assembly
Transcription initiation factor IIA subunit 2/TraesCS1D02G024600	Transcription initiation from RNA polymerase II promoter
MYB transcription factor 80/TraesCS2A02G206400	Transcription regulatory region DNA binding, cell differentiation
MYB transcription factor 74/TraesCS2D02G209600	
MIKC-type MADS-box transcription factor WM30/TraesCS2A02G337900	DNA binding, transcription factor activity, protein dimerization activity, RNA polymerase II regulatory region
MYB transcription factor SM152-3/TraesCS7A02G179900	Transcription regulatory region DNA binding, cell differentiation
MYB transcription factor SM152-1/TraesCS7B02G085100	
MYB transcription factor SM152-2/TraesCS7D02G181400	
MYB13 transcription factor/TraesCS3A02G535100	
R2R3 MYB transcriptional factor/TraesCS7D02G272400	
NAC transcription factor 6A/TraesCS5B02G054200	DNA binding, transcription regulation
NAC transcription factor/TraesCS5D02G059700	
MYB-related protein/TraesCS3D02G540600	Sequence-specific DNA binding, transcription regulation
MYB protein/TraesCS3B02G612200	
R2R3-MYB protein/TraesCS3A02G108000	Sequence-specific DNA binding, cell differentiation
NAC domain-containing protein 2a-like protein/TraesCS5B02G480900	Sequence-specific DNA binding, transcription regulation
NAC transcription factor 6A/TraesCS3A02G406000	
NAC transcription factor 6B/TraesCS3B02G439600	
NAC domain-containing protein 18/TraesCS7D02G263800	
RNAC1 transcription factor/TraesCS2D02G324700	
WRKY transcription factor/TraesCS3B02G324400	
WRKY80 transcription factor/TraesCS6A02G146900	
**Auxin**	
Auxin-responsive protein/TraesCS4B02G070300	Uncharacterized protein
Auxin-responsive protein/TraesCS4A02G245100	
Auxin-responsive protein/TraesCS4D02G069100	
Auxin-responsive protein/TraesCS5D02G069300	
Auxin-responsive protein/TraesCS5B02G058500	
Auxin-responsive protein/TraesCS5D02G392000	
Auxin-responsive protein/TraesCS5A02G382600	Auxin-induced protein, auxin-activated signaling pathway
Auxin-responsive protein/TraesCS5A02G058700	
Auxin-responsive protein/TraesCS5B02G386800	Uncharacterized protein
Auxin-responsive protein/TraesCS5D02G069200	Auxin-induced protein, auxin-activated signaling pathway
Auxin efflux carrier component/TraesCS7B02G095500	Auxin efflux carrier, auxin-activated signaling, transmembrane transport
Auxin-responsive protein/TraesCS5D02G388200	Auxin-responsive protein
Auxin-responsive protein/TraesCS7B02G256100	Uncharacterized protein
Auxin-responsive protein/TraesCS7A02G371500	
Auxin-responsive protein/TraesCS5B02G381800	
**Cytokinin**	
Cytokinin riboside 5′-monophosphate phosphoribohydrolase/TraesCS3A02G251500	Hydrolase activity cytokinin, biosynthetic process
**Mitogen-activated Protein Kinase**	
Mitogen-activated protein kinase/TraesCS7A02G422500	ATP binding, cell division, response to abscisic acid, ethylene, hydrogen peroxide, priming of cellular response to stress
Mitogen-activated protein kinase/TraesCS7A02G111300	
**Abscisic Acid**	
HVA22-like protein/TraesCS3A02G283300	Receptor expression-enhancing protein
HVA22-like protein/TraesCS4A02G080700	
Abscisic stress-ripening protein/TraesCS4D02G109500	
HVA22-like protein/TraesCS2B02G313000	Uncharacterized protein
HVA22-like protein/TraesCS2D02G294700	
HVA22-like protein/TraesCS2A02G296800	Receptor expression-enhancing protein
**Ethylene**	
Transmembrane 9 superfamily member/TraesCS6D02G265500	Multi-pass membrane protein, signal peptide, protein localization to membrane
Transmembrane 9 superfamily member/TraesCS6B02G313900	
Ethylene receptor/TraesCS6A02G399400	Ethylene binding, signaling pathway
**Antioxidative Enzymes**	
Superoxide dismutase/TraesCS2A02G537100	Catalyze the conversion of superoxide radicals to molecular oxygen, metal ion binding, stress responses
Catalase/TraesCS7B02G473400	Heme binding, metal ion binding, reactive oxygen species detoxification, hydrogen peroxide catabolic process, response to abscisic acid and salicylic acid
Catalase/TraesCSU02G105300	
Dopamine beta-monooxygenase/TraesCS3B02G300400	Oxidation–reduction process, metal ion binding, electron transport, flavin adenine dinucleotide binding, auxin biosynthesis process, NADP binding
Glutathione reductase/TraesCS6B02G423100	Oxidoreductase activity, detoxification of ROS, glutathione metabolism
Superoxide dismutase [Cu–Zn]/TraesCS4D02G242800	Catalyze the conversion of superoxide radicals to molecular
Flavin-containing monooxygenase/TraesCS4B02G370200	Oxidation–reduction process, flavin adenine dinucleotide binding, NADP binding, auxin biosynthetic process
Flavin-containing monooxygenase/TraesCS1A02G211100	
Flavin-containing monooxygenase/TraesCS5B02G531000	
Flavin-containing monooxygenase/TraesCS4A02G313200	
Flavin-containing monooxygenase/TraesCS7D02G538300	
Flavin-containing monooxygenase/TraesCS7A02G552000	
Flavin-containing monooxygenase/TraesCS2D02G012100	
Flavin-containing monooxygenase/TraesCS2B02G010100	
Flavin-containing monooxygenase/TraesCS2A02G011500	
Flavin-containing monooxygenase/TraesCS4B02G366800	
Flavin-containing monooxygenase/TraesCS4D02G360900	
Flavin-containing monooxygenase/TraesCS5A02G534500	
Flavin-containing monooxygenase/TraesCS7D02G538700	
Flavin-containing monooxygenase/TraesCS5D02G355700	
Flavin-containing monooxygenase/TraesCS5B02G350700	
Flavin-containing monooxygenase/TraesCS3A02G010900	
Flavin-containing monooxygenase/TraesCS5A02G349300	
Flavin-containing monooxygenase/TraesCS4D02G269000	

## Supplementary Materials

The following are available online at https://www.mdpi.com/2223-7747/8/12/584/s1, Figure S1: Transcription factors (187 genes), Figure S2: MYB (A, 38 genes), NAC (B, 15 genes), and WRKY (C, 18 genes) transcription factors, Figure S3: Auxins-related genes (195 genes), Figure S4: Cytokinin (A, 40 genes), gibberellin (B, 3 genes), zeatin epoxidase (C, 3 genes), mitogen-activated protein kinase (D, 40 genes) related genes, Figure S5: Abscisic acid (A, 50 genes), ethylene (B, 68 genes) related genes, Figure S6: Antioxidative enzyme (156 genes) related genes, Figure S7: Root box-pin board set, Figure S8: Root-basket set.
