# Supplementary material for: Variation Among Spring Wheat (Triticum aestivum L.) Genotypes in Response to the Drought Stress. II—Root System Structure
Source: Plants (Basel). 2019 Dec 8;8(12):584. doi: 10.3390/plants8120584 (PMC6963452; doi:10.3390/plants8120584)
Supplement: Supplementary file 1 [file plants-08-00584-s001.pdf]

Supplementary

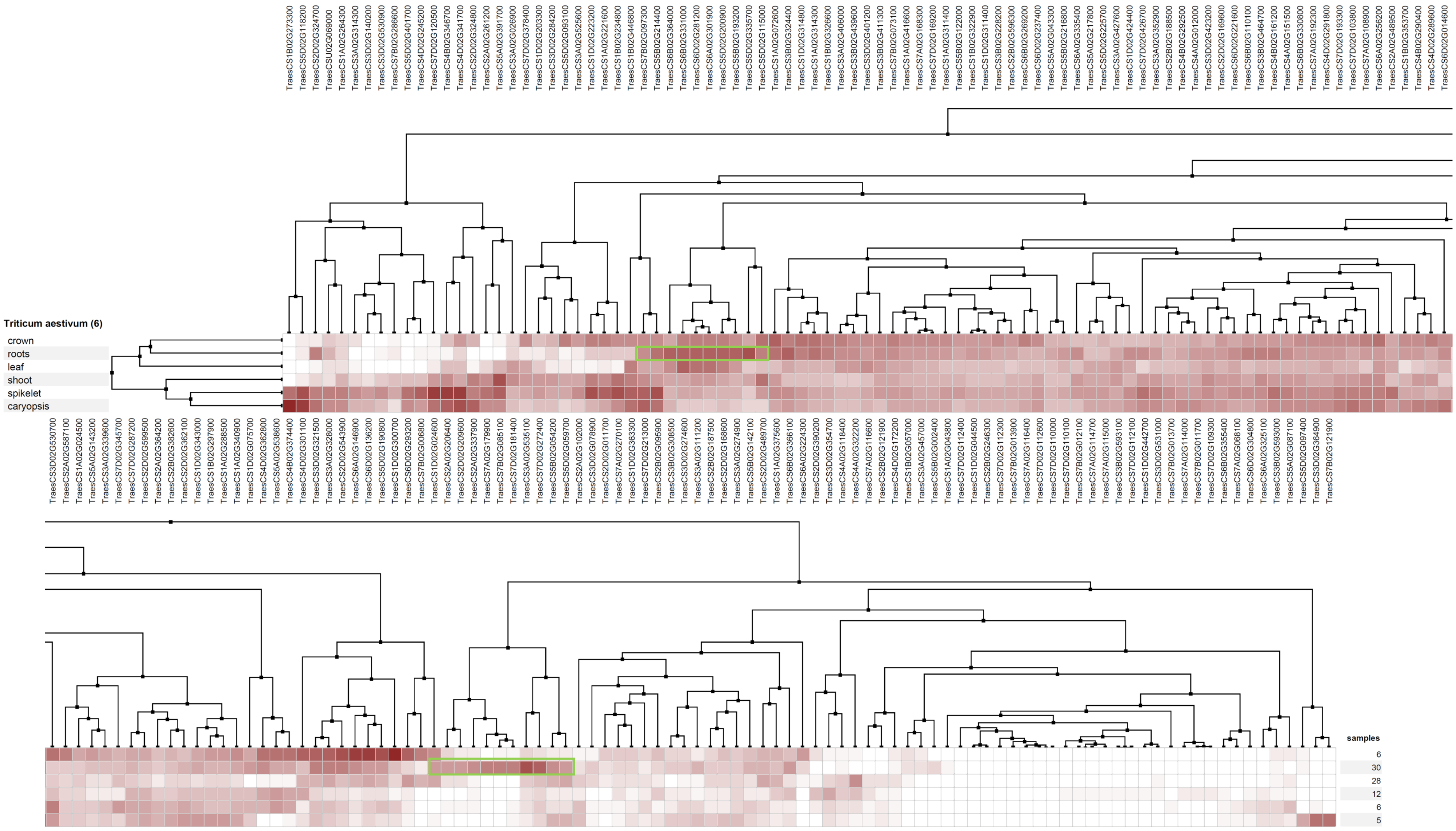

Figure S1. Transcription factors (187 genes). Transcripts were analyzed on the platform TA_mRNASeq_WHEAT_GL-1. Percentage of expression potential: 0, 20, 40, 60, 80, 100.Green boxes indicate selected genes described in Table 6.

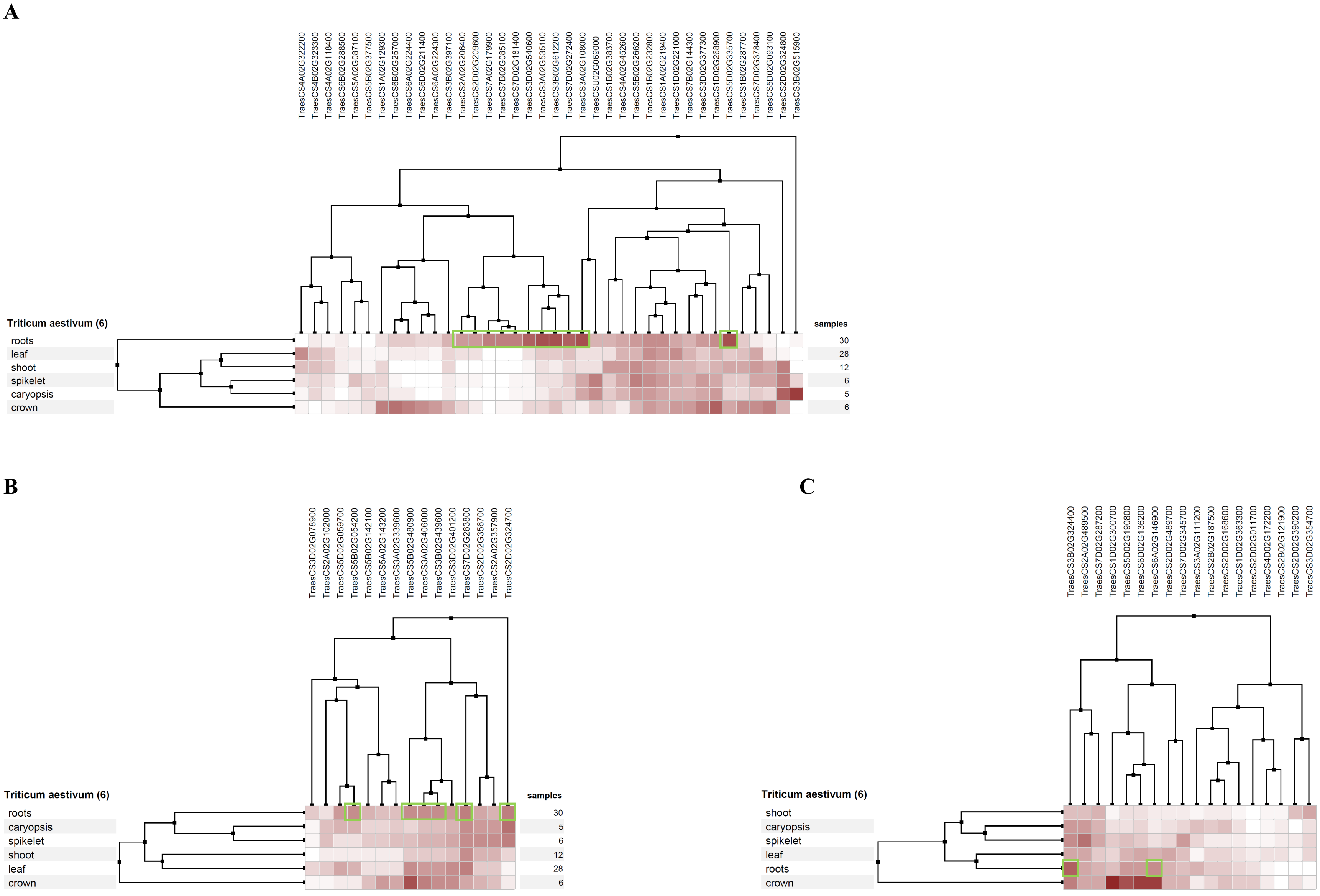

Figure S2. MYB (**A**, 38genes), NAC (**B**, 15 genes), and WRKY (**C**, 18 genes) transcription factors. Transcripts were analyzed on the platform TA_mRNASeq_WHEAT_GL-1. Percentage of expression potential: 0, 20, 40, 60, 80, 100. Green boxes indicate selected genes described in Table 6.

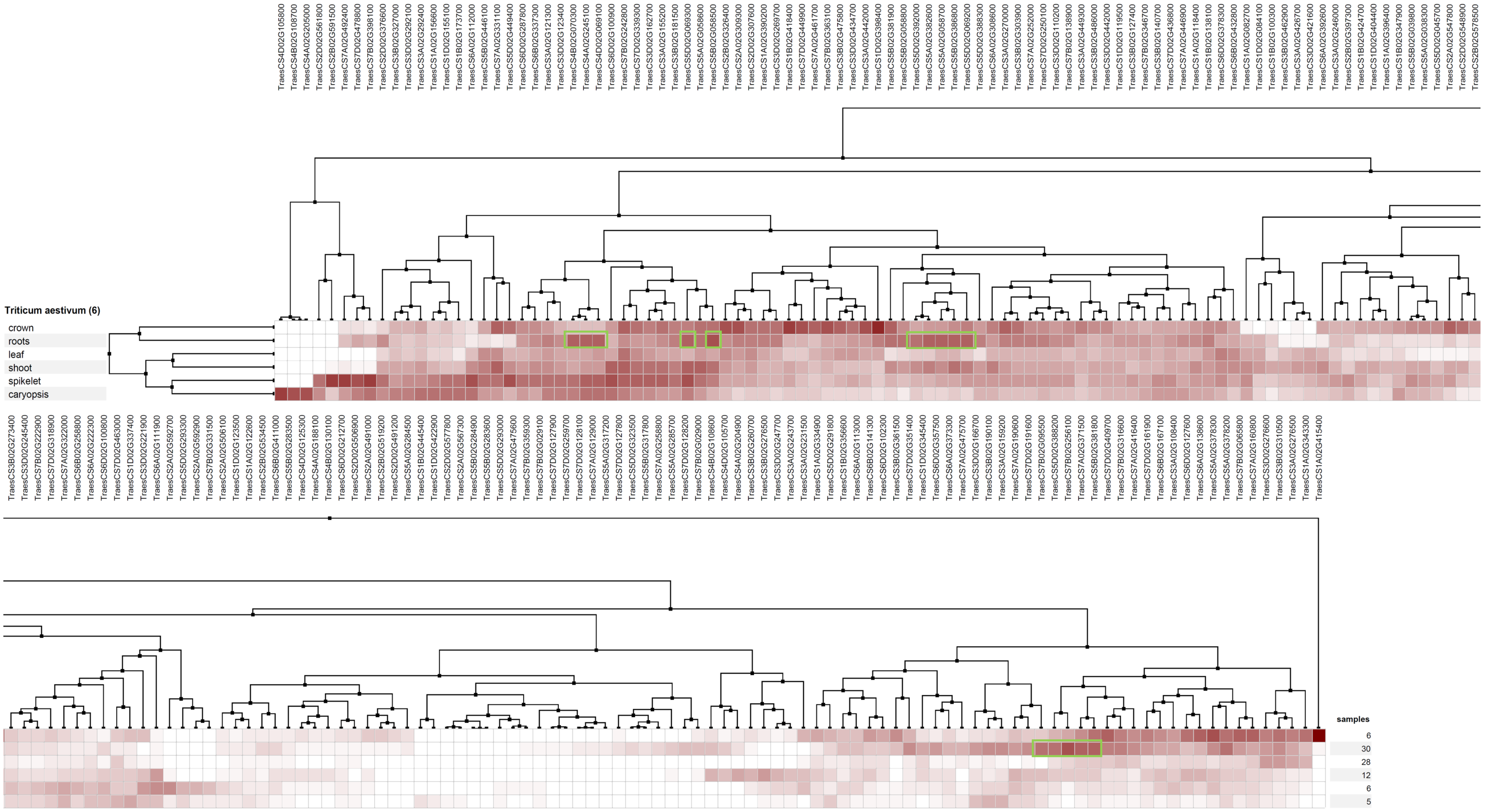

Figure S3. Auxins-related genes (195 genes). Transcripts were analyzed on the platform TA_mRNASeq_WHEAT_GL-1. Percentage of expression potential: 0, 20, 40, 60, 80, 100. Green boxes indicate selected genes described in Table 6.

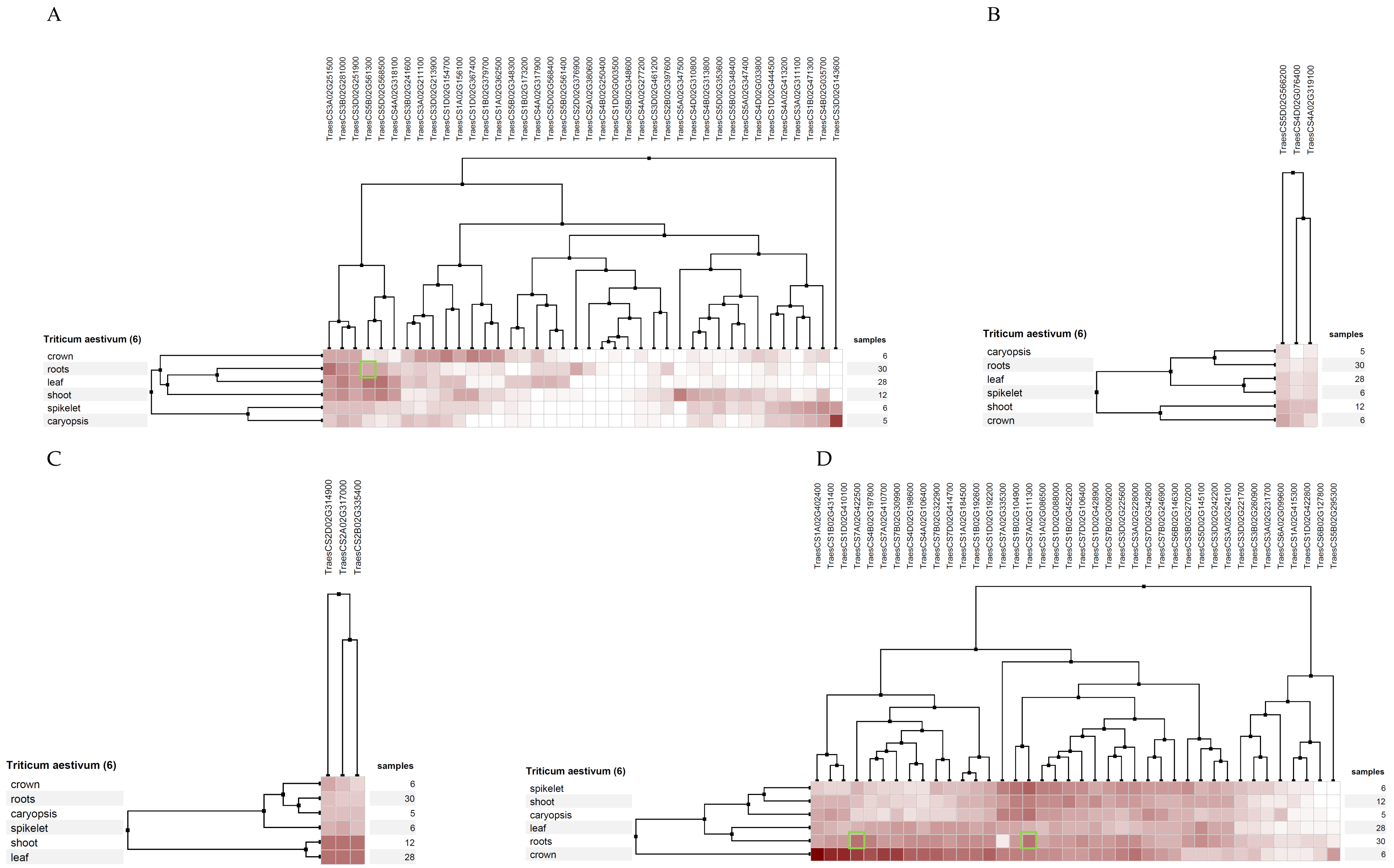

Figure S4. Cytokinin (**A**, 40 genes), gibberellin (**B**, 3 genes), zeatin epoxidase (**C**, 3 genes), mitogen-activated protein kinase (**D**, 40 genes) related genes. Transcripts were analyzed on the platform TA_mRNASeq_WHEAT_GL-1. Percentage of expression potential: 0, 20, 40, 60, 80, 100. Green boxes indicate selected genes described in Table 6.

A

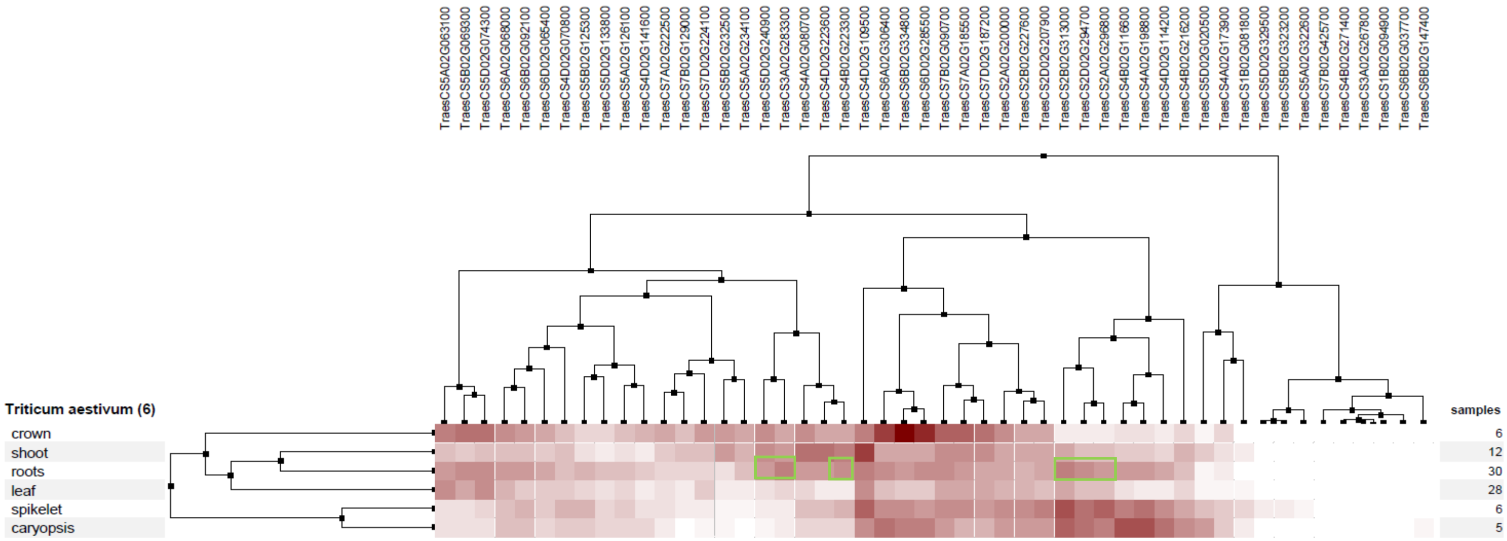

B

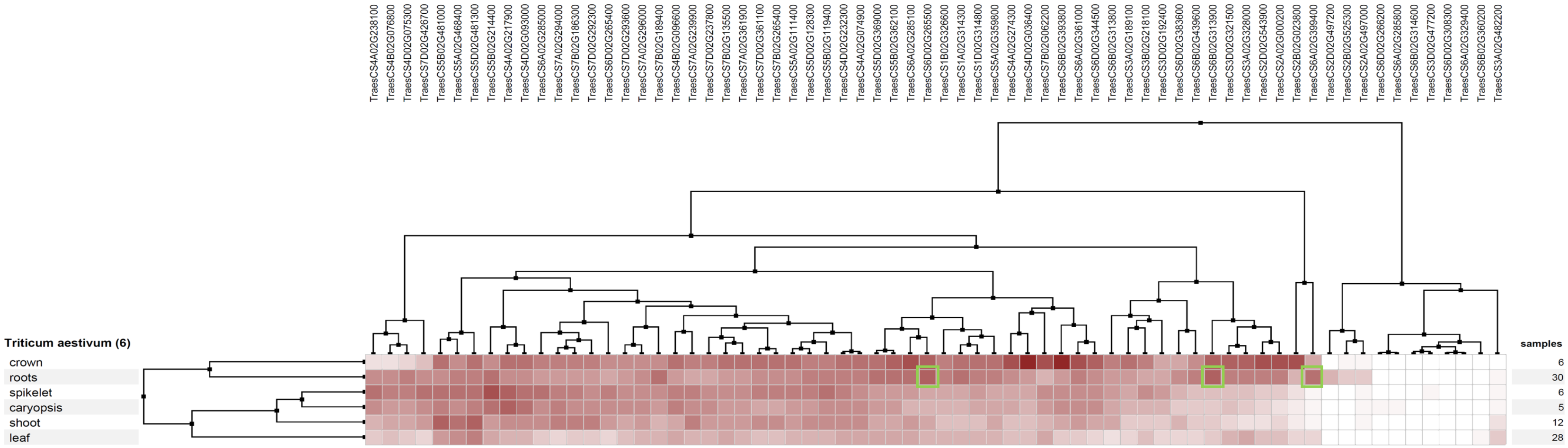

Figure S5. Abscisic acid (**A**, 50 genes), ethylene (**B**, 68 genes) related genes. Transcripts were analyzed on the platform TA_mRNASeq_WHEAT_GL-1. Percentage of expression potential: 0, 20, 40, 60, 80, 100. Green boxes indicate selected genes described in Table 6.

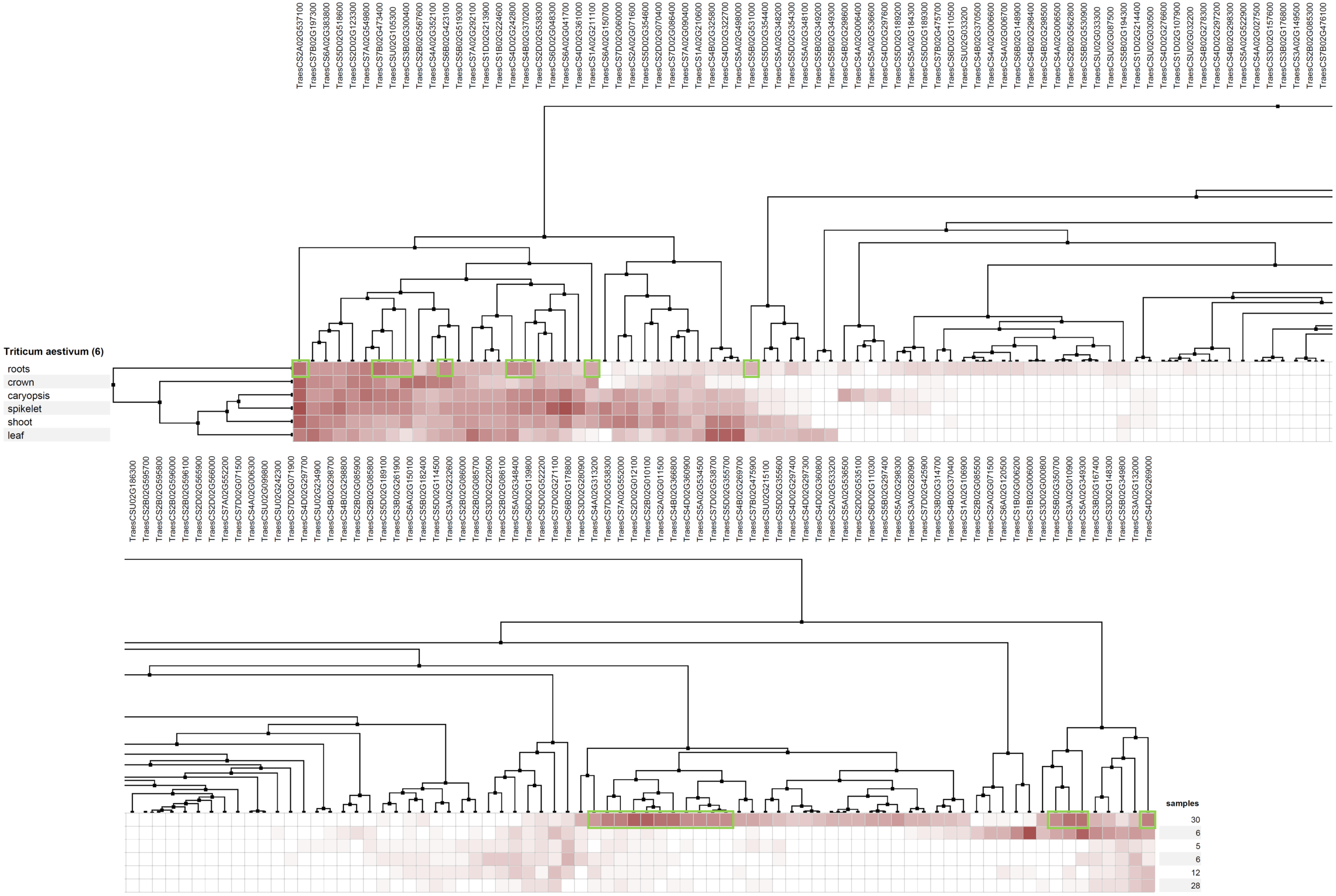

Figure S6. Antioxidative enzyme (156 genes) related genes. Transcripts were analyzed on the platform TA_mRNASeq_WHEAT_GL-1. Percentage of expression potential: 0, 20, 40, 60, 80, 100. Green boxes indicate selected genes described in Table 6.

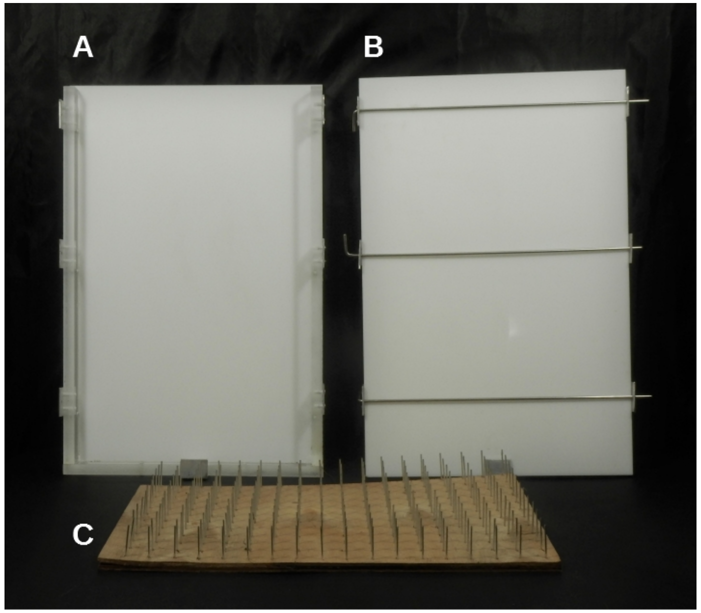

Figure S7. Root box-pin board set: open root-box (**A**); closed root-box (**B**); the pin-board (**C**).

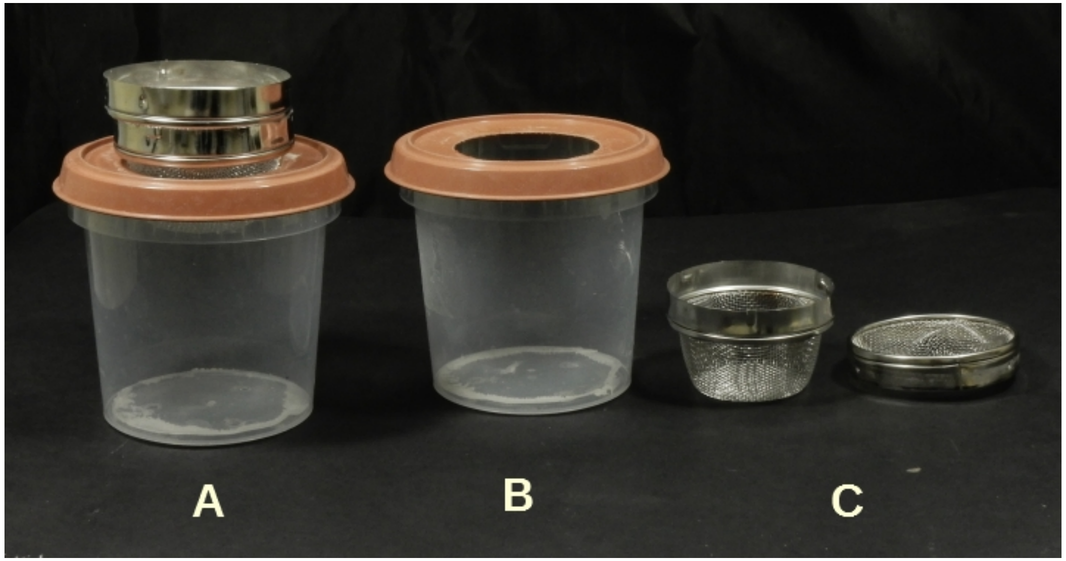

Figure S8. Root-basket set (**A**): pot with mounting plate (**B**); lower and upper basket (**C**).closed root-box (**B**); the pin-board (**C**).
